# Increased infiltration of M2-polarized tumour-associated macrophages is highly associated with advanced disease stage and high expression of PD-L1 in buccal mucosa carcinoma

**DOI:** 10.1007/s12672-024-01190-y

**Published:** 2024-07-29

**Authors:** Hao-Jia Sun, Zhui-Feng Zheng, Li-Jun Zhang, Le Fang, Hua Fu, Shao-Yang Chen, Rong-Xiu Feng, Xiao-Yang Liu, Qing-Nan Tang, Xue-Wen Liu

**Affiliations:** 1 Department of Oncology, Third Xiangya Hospital, Central South University, Changsha, 410013 Hunan China; 2 Department of Breast Medical Oncology, Fujian Cancer Hospital and the Fujian Medical University Cancer Hospital, Fuzhou, 350014 Fujian China; 3 Department of Oncology, Huaihua First People’s Hospital, Huaihua, 418000 Hunan China; 4 Department of Oncology, Loudi Central Hospital, Loudi, 417099 Hunan China; 5 Department of Pathology, Third Xiangya Hospital, Central South University, Changsha, 410013 Hunan China; 6 Department of Radiation Oncology, Xiangtan Central Hospital, Xiangtan, 411199 Hunan China; 7 Department of Oncology, Changde First People’s Hospital, Changde, 415003 Hunan China

**Keywords:** Buccal mucosa carcinoma, Tumour-associated macrophages, PD-L1, EGFR, CD8+ T cells

## Abstract

**Objective:**

To assess the infiltration characteristics of tumour-associated macrophages (TAMs) in buccal mucosa carcinoma (BMC) and the correlation of these features with clinicopathological factors.

**Materials and methods:**

Immunohistochemistry was used to detect the expression of TAM-related markers (CD68, CD163, CD206), CD8+ T cell markers, PD-L1, and epidermal growth factor receptor (EGFR) in 46 patients with mucosal cancer after radical surgery. In addition, the correlation between TAM infiltration and clinical characteristics, PD-L1 expression, and EGFR expression was analysed.

**Results:**

A high infiltration level of M2-polarized (CD206+) TAMs and M2-polarized (CD163+) TAMs was more common in stage T3–T4, N+, III–IV patients than in other patient groups (P < 0.05). The infiltration degree of M2-polarized (CD68+) TAMs was positively correlated with the PD-L1 TPS (P = 0.0331). The infiltration level of M2-polarized (CD206+) TAMs was higher in the EGFR high expression group than in the EGFR low expression group (P = 0.040).

**Conclusion:**

High infiltration of M2-polarized TAMs is highly associated with advanced disease stage and higher expression of PD-L1 and EGFR in BMCs, suggesting that M2-polarized TAMs infiltration can serve as a potential therapeutic target.

**Supplementary Information:**

The online version contains supplementary material available at 10.1007/s12672-024-01190-y.

## Introduction

Nowadays, the oral squamous cell carcinoma (OSCC) is one of the most common malignant tumours [1]. According to global cancer statistics in 2020, oral cancer accounted for 2.0% and 1.8% of global cancer cases and related deaths, respectively [2], and these rates have shown an overall upwards trend in recent years [3]. It is estimated that there will be ~ 600,000 new cases of oral cancer by 2030 [4]. Due to the habit of chewing betel nut, the buccal mucosa is the most common site of oral cancer in Hunan Province of China [5]. Buccal mucosal carcinoma (BMC) is a type of OSCC, accounting for ~ 5–10% of OSCC cases [6]. However, a few clinical studies have specifically focused on BMC, and most reports were limited because of a small sample size [7, 8].

Immunotherapy is a new treatment for BMC in recent years. Pembrolizumab alone or combined with chemotherapy has been approved as the first-line treatment for recurrent and metastatic OSCC [9]. The approval of this drug has encouraged more attention on the immune micro-environment and its relation to the efficacy of immunotherapy.

Tumour associated macrophages (TAMs) are the most abundant components in the tumour immune micro-environment. Under the stimulation of different specific cytokines, they can be polarized into M1 and M2 phenotypes. In general, M1-polarized TAMs act in tumour-inhibiting manner, and M2-polarized TAMs act in tumour-promoting manner. According to the stimulation of a variety of different cytokines, M2-polarized TAMs can be divided into four subtypes: M2a, M2b, M2c and M2d. All four subtypes of M2-polarized TAMs can be identified by the specific surface biomarker CD206, while CD163 is a specific biomarker of M2c-polarized TAMs [10, 11]. Two independent studies proved that TAMs are the main immune cells expressing PD-L1 in the interstitial region of hepatocellular carcinoma firstly [12, 13]. These PD-L1 + TAMs were activated by tumour-derived interleukin-10 (IL-10), which can mediate the dysfunction of CD8+ T cells through PD-1/PD-L1 interaction. Shima et al. [14] found that when lung cancer cell lines were cocultured with M2-polarized TAMs, the expression level of human lung cancer cell lines A549 and H1975 was increased remarkably, and inhibition of transforming growth factor-β (TGF-β) decreased PD-L1 expression to the baseline level. Similar results were also found in head and neck squamous cell carcinoma, cholangiocarcinoma as well as bladder cancer [15–18]. Multiple previous studies revealed that regulating the polarization of TAMs to promote the M1 phenotype could reverse the immunosuppressive effect [19–21]. These studies suggest that agents targeting M2-polarized TAMs combined with immune checkpoint inhibitors may also be an effective strategy for treatment of malignant tumours [22].

In this study, to reveal the role of M2-polarized TAMs and to provide a reference for prospective studies on targeting TAMs in BMC, we summarized the characteristics of M2-polarized TAMs infiltration in BMC and evaluated the correlations between the M2-polarized TAMs infiltration and clinical characteristics, the expression of PD-L1 and epidermal growth factor receptor (EGFR).

## Materials and methods

### Patients collection

The study has met with approval by the Ethics Committee of Hunan Cancer Hospital, the Affiliated Cancer Hospital of Xiangya School of Medicine/Central South University. BMC patients were enrolled based on specific collection criteria at Hunan Cancer Hospital from 2016.01 to 2018.12. Patients’ clinical data, including name, gender, age, pathological characteristics, imaging and TNM stage, were retrospectively collected. The inclusion criteria were as follows: (1) age older than 18 years old and pathological diagnosis of squamous cell carcinoma of buccal mucosa; (2) complete routine preoperative examination and systemic evaluation to exclude distant metastasis; (3) surgery as the first treatment; (4) acceptance of completely primary tumour resection and lymph node dissection; (5) treatment with postoperative adjuvant chemotherapy and radiotherapy, if necessary; and (6) complete follow-up data. The exclusion criteria were as follows: (1) concurrent other primary malignant tumours; (2) presence of other severe organic diseases and immune diseases; (3) below-standard quality of pathological specimens or lack of specimens; (4) previous chemotherapy or radiation treatment; and (5) recurrence within 3 months after radical operation. Finally, 46 patients were selected from 613 patients with mucosal cancer and included in this study, and the clinical characteristics of the 46 patients with BMC are shown in Table 1.

**Table 1:** Pathological characteristics of 46 patients with BMC

Pathological features	Number	Percentage (%)
All cases	46	100
Age		
< 49 years old	22	47.8
≥ 49 years old	24	52.2
Gender		
Male	42	91.3
Female	4	8.7
T stage		
T1–T2	39	84.8
T3–T4	7	15.2
N stage		
N0	37	0.804
N1–N2	9	19.6
Clinical stage		
I–II	34	73.9
III–IV	12	26.1
Pathological stage		
Well differentiated	33	71.7
Poorly differentiated	13	18.3

### TNM staging and pathological grading

All tumours were restaged according to the latest Union for International Cancer Control (UICC) 8th edition TNM staging method for oral cancer. T and N stages were determined by medical imaging data and postoperative pathological reports; M stages were determined by the medical imaging data in the Electronic medical record system. Subsequently, tumours were then divided to T1–2 and T3–T4 groups, N0 and N + groups, and stage I–II and stages III–IV groups. According to the postoperative histopathological data, tumours were divided into highly differentiated group and poorly differentiated group.

### Immunohistochemistry

The immunohistochemistry were performed to evaluate TAM distribution, PD-L1 expression, and EGFR expression of the BMC tissues using the Ventana BenchMar GX system (Roche/Ventana Medical Systems, Tucson, USA). Formalin-fixed paraffin-embedded BMC tissues of enrolled patients were collected and then cut into 5 μm sections and fitted on slides. Dewaxing tissue sections with xylene and alcohol. In order to obtain antigens from the tissues, they were placed in 0.01 M citrate buffer (pH 6.0) and subjected to microwave heating. Add 1% potassium permanganate for 10 min to inactivate endogenous enzymes. The primary antibodies were incubated at 4 °C overnight. After being washed by phosphate buffered saline, sections were incubated with the second antibody at 37 °C for 30 min.Add diaminobenzidine to sections for color development, followed by hematoxylin counterstaining and alcohol dehydration. The following antibodies were used: anti-CD68 (KP1, Abcam, 1:200), anti-CD163 (epr19518, Abcam, 1:200), anti-CD206 (epr22489-7, Abcam, 1:200), anti-PD-L1 (22C3, Dako, 1:200), and anti-EGFR (Rma-0804egfr, Maixin company, Fuzhou, Fujian, China, 1:200). Two pathologists independently evaluated the results of staining.

### Evaluation of immunohistochemistry

Positive staining of the membrane or cytoplasm of macrophages was indicated by yellow or brown staining. We evaluated the results of IHC staining of the tumour area, para-carcinoma tissue area, and normal tissue area, respectively. First, 1 hot spot area (positive staining-dense area) was selected under the low magnification (× 100). Next, 5 staining hot spots were selected under the high microscope (× 400) for observation, and the number of positively stained cells was counted in each high-power field; then, the average number of positive cells in the 5 fields was calculated. Finally, the median number of CD68, CD163, and CD206 positive macrophages in the tumour area of BMC was calculated. Samples with a positive macrophage number greater than the median number were included in the high expression group.

T lymphocyte cell membrane staining of yellow or brown indicated positive expression.

First, under the low magnification (× 100), 1 staining hot spot was selected from the tumour area, the para-carcinoma area, and the normal tissue area. Next, under the high magnification (× 400), 5 staining hot spots were selected, and the number of positive cells among 100 cells was determine. The average number of CD8+ T lymphocytes in 5 hot spots was calculated. Then, the average was divided by 100 to obtain the CD8+ T lymphocyte density. Finally, the median of CD8+ T lymphocyte density was calculated, and samples with density greater than the median value were considered to have significant infiltration.

The expression level of EGFR was determined based on the proportion (out of 100) of normal epithelial cells or tumour cells with positive membrane staining under high magnification. EGFR expression was classified as positive if ≥ 1% of the cell membrane demonstrated membranous staining and was otherwise classified as negative. Samples with EGFR expression in the tumour area ≥ median were considered to have high EGFR expression.

The expression of PD-L1 on tumour and immune cells was analysed using the tumour cell proportion score (TPS) and immune cell proportion score (IPS), respectively. If TPS or IPS ≥ 1%, PD-L1 expression was considered positive. The combined positive score (CPS) referred to the number of PD-L1-stained cells (tumour cells, lymphocytes, and macrophages) divided by the total number of viable tumour cells multiplied by 100. CPS ≥ 1 was considered PD-L1 positive.

### Statistical analysis

SPSS software (v26.0) was used to analyse the data. Chi-square test was used to analyse the relationships among the M2-polarized TAMs infiltration and the clinicopathological characteristics, the CD8+ T-cell infiltration level, and the expression of EGFR. The correlation between M2-polarized TAMs infiltration level and PD-L1 TPS, IPS, and CPS was explored through t test. Two-tailed P < 0.05 was considered to indicate statistical significance. To judge the ability of M2-polarized TAMs infiltration levels to distinguish BMC, the receiver operating characteristic (ROC) curves were drawn by GraphPad Prism (v8.3.0). By calculating the area under the ROC curve, the diagnostic value in M2-polarized TAMs infiltration between BMC and para-carcinoma tissue was further verified. Multivariate logistic regression was employed to identify the independent risk factors of M2-polarized TAMs infiltration level in BMC tissues. There was statistical significance when p < 0.05.

## Results

### The expression of M2-polarized TAMs markers in BMC

**Fig. 1:**
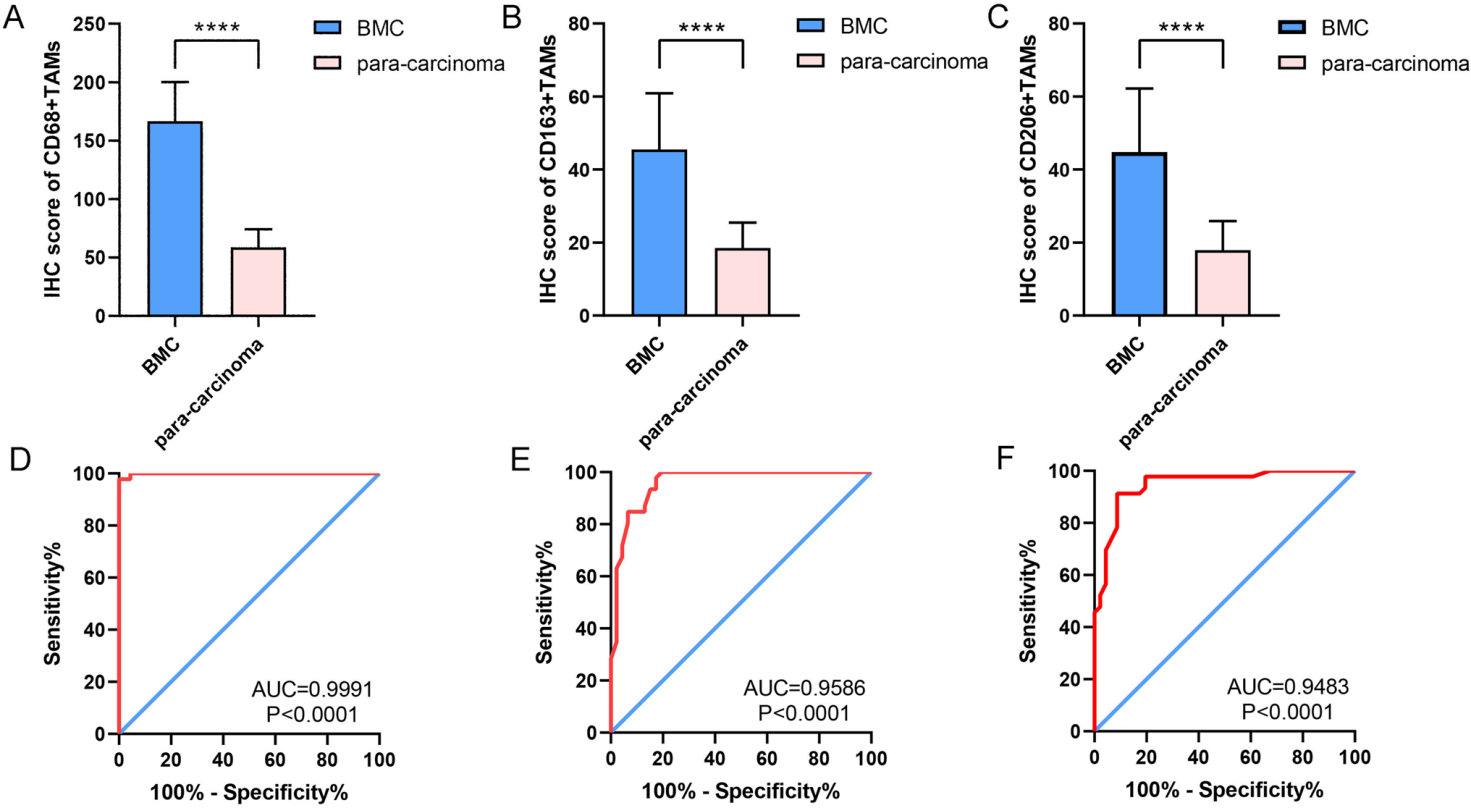
Infiltration of M2-polarized TAMs in different regions of BMC. **A** M2-polarized (CD68+) TAMs in BMC. **B** M2-polarized (CD163+) TAMs in BMC. **C** M2-polarized (CD206+) TAMs in BMC. **D** ROC curve of CD68+TAMs Infiltration in BMC. **E** ROC curve of CD163+TAMs Infiltration in BMC. **F** ROC curve of CD206+TAMs Infiltration in BMC

**Fig. 2:**
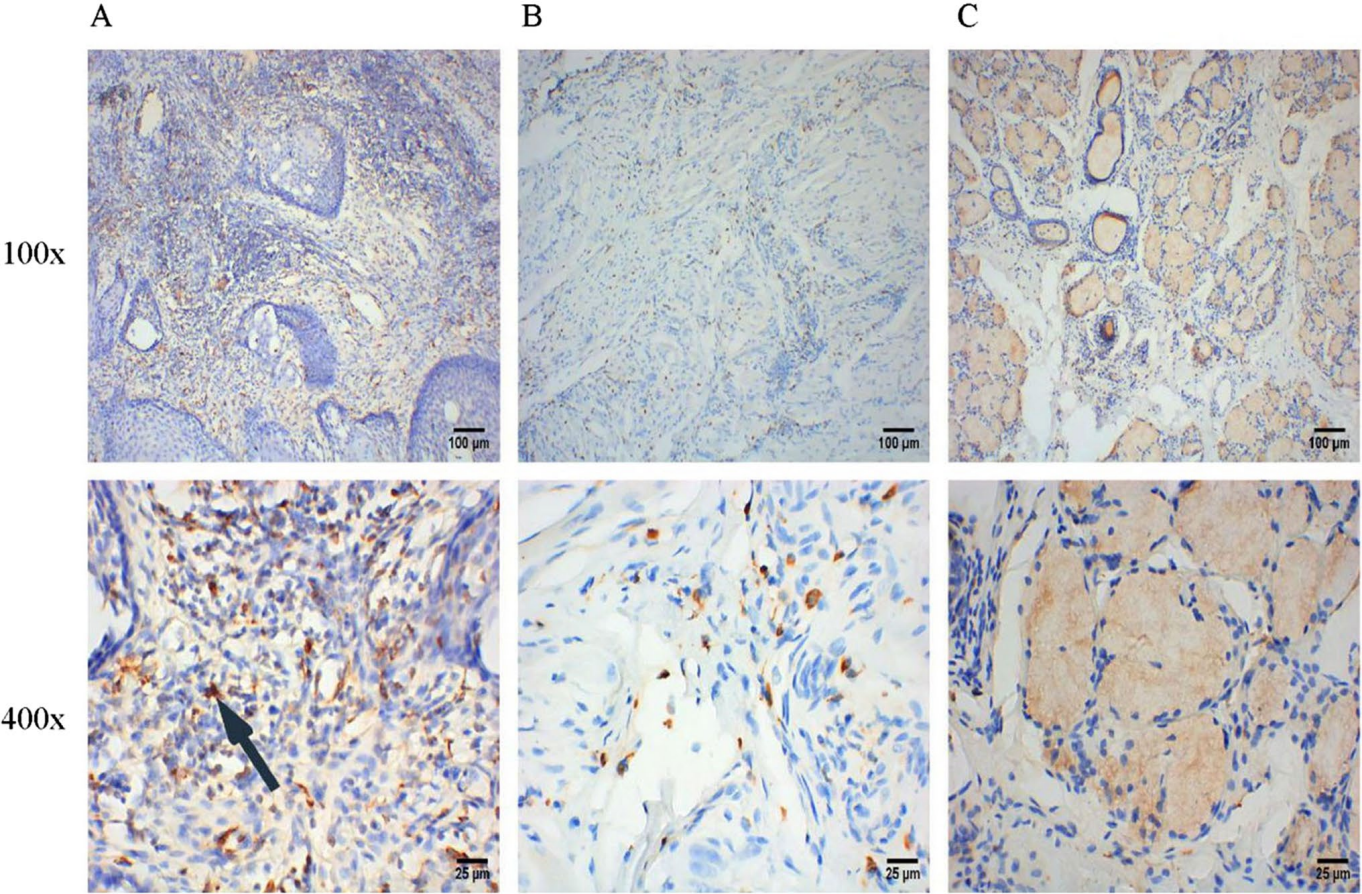
IHC staining of M2-polarized (CD68+) TAMs in different tissues. **A** IHC staining of M2-polarized (CD68+) TAMs in BMC tissues. **B** IHC staining of M2-polarized (CD68+) TAMs in para-carcinoma tissues. **C** IHC staining of M2-polarized (CD68+) TAMs in normal buccal mucosal tissues

**Fig. 3:**
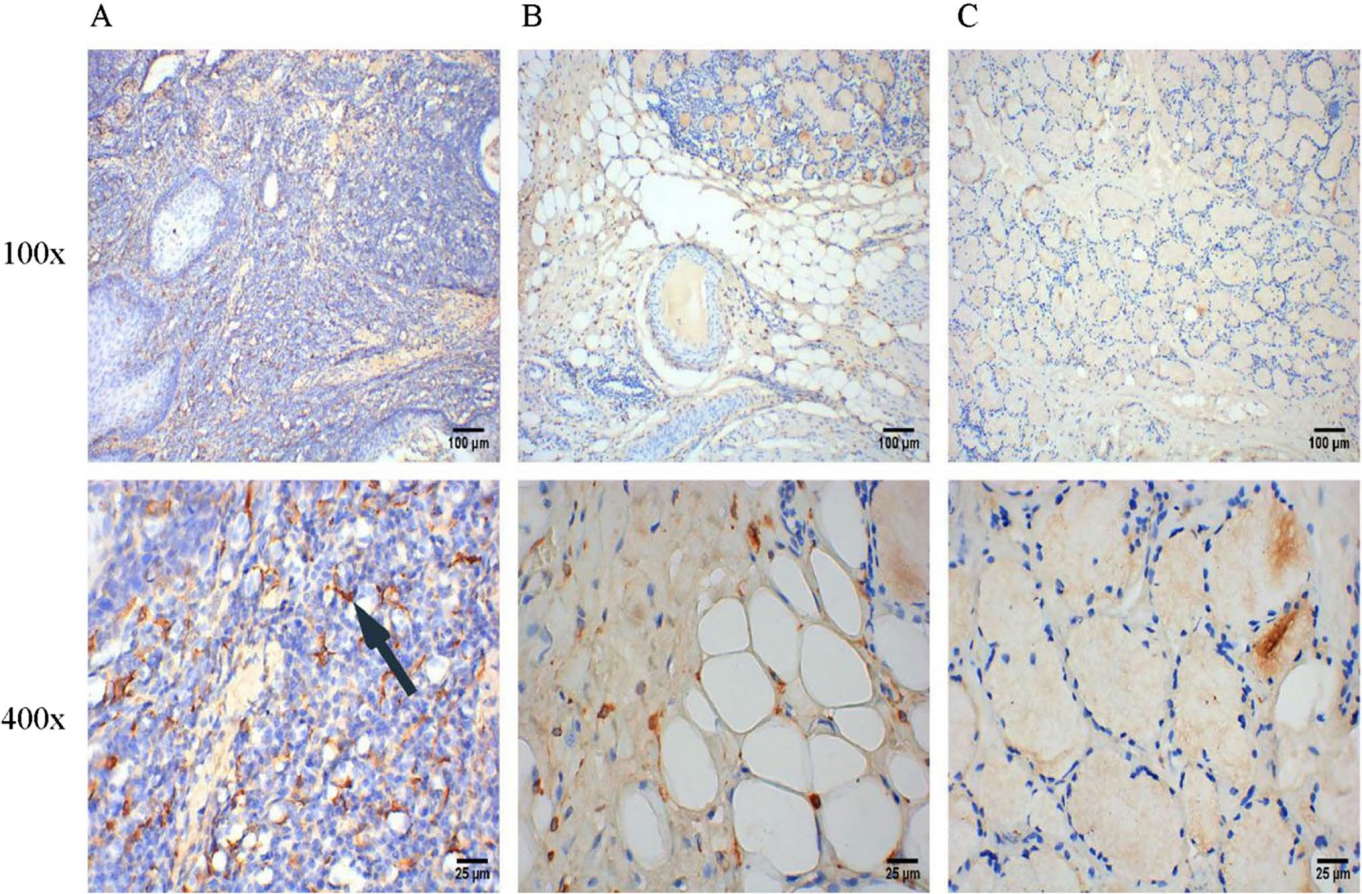
IHC staining of M2-polarized (CD163+) TAMs in different tissues. **A** IHC staining of M2-polarized (CD163+) TAMs in BMC tissues. **B** IHC staining of M2-polarized (CD163+) TAMs in para-carcinoma tissues. **C** IHC staining of M2-polarized (CD163+) TAMs in normal buccal mucosal tissues

**Fig. 4:**
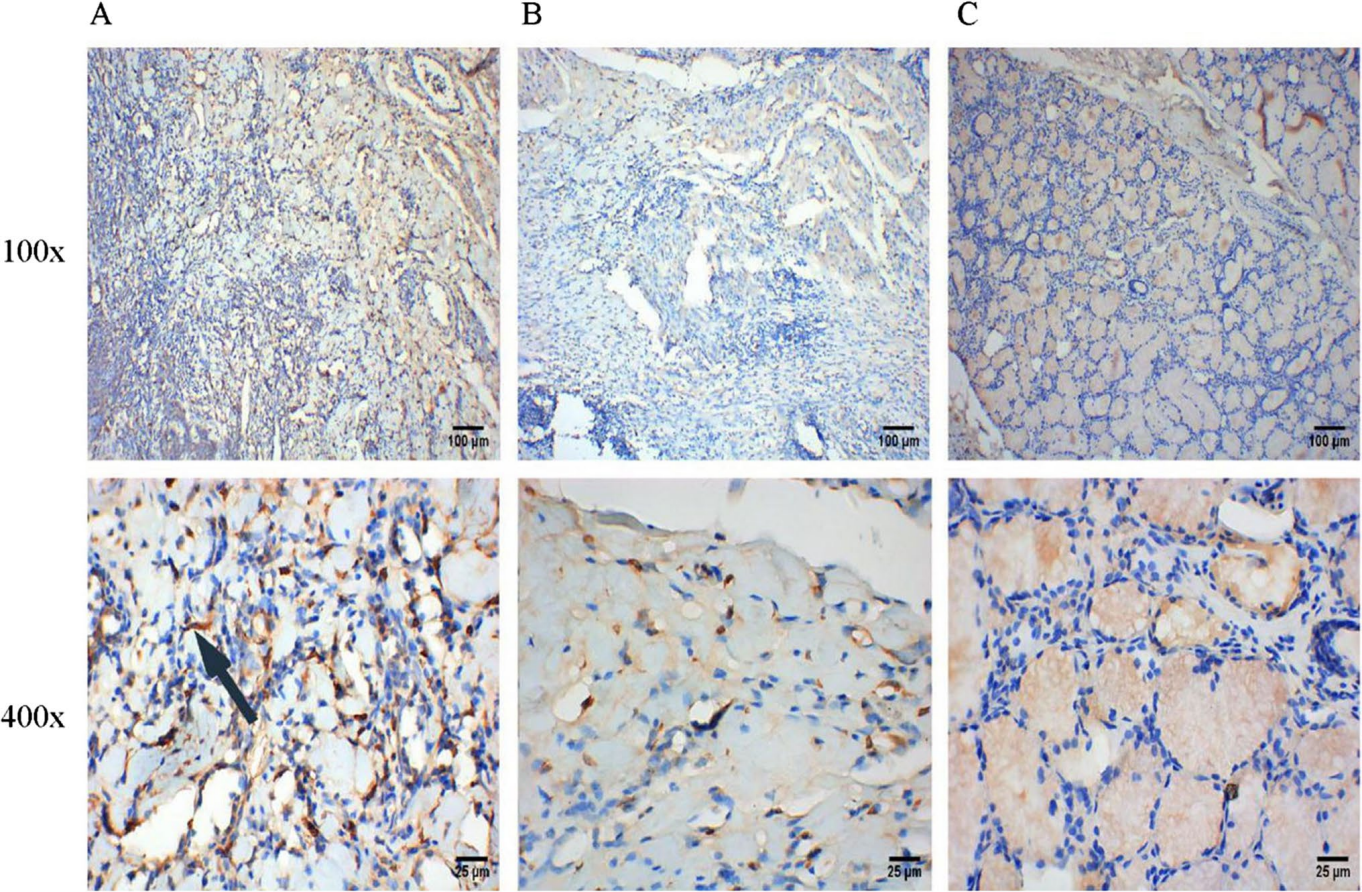
IHC staining of M2-polarized (CD206+) in different tissues. **A** IHC staining of M2-polarized (CD206+) TAMs in BMC tissues. **B** IHC staining of M2-polarized (CD206+) TAMs in para-carcinoma tissues. **C** IHC staining of M2-polarized (CD206+) TAMs in normal buccal mucosal tissues

The expression of M2-polarized TAMs markers (CD68+, CD163+ and CD206+) was detected by IHC, and the results are shown in the scatter plots in Fig. 1. The expression of CD68+ in BMC tissues were significantly higher than those in the group of normal buccal mucosal tissues (Fig. 1A, P < 0.05). The results of ROC curves showed that the AUCs of all ROC curves were greater than 0.94 (P < 0.0001, Fig. 1D–F), which suggested the great diagnostic value of M2-polarized TAMs in BMC. As shown in Fig. 2, M2-polarized (CD68+) TAMs are densely distributed in BMC tissues, scattered in adjacent tissues, and have no significant infiltration in the normal group. In addition, the expression levels of CD163 + and CD206 + in BMC tissues were also significantly increased compared to those in normal buccal mucosal tissues (Fig. 1B, C, P < 0.05). The IHC staining results for CD163 + and CD206 + in different tissues are illustrated in Figs. 3 and 4.

### Correlation between the infiltration of M2-polarized TAMs and clinical features

As shown in Table 2, the infiltration level of M2-polarized (CD68+ and CD206+) TAMs was correlated with sex (P < 0.05). In addition, the infiltration of M2-polarized (CD163 +) TAMs was positively correlated with T stage (P = 0.006), lymph node metastasis (P = 0.014), and clinical stage (P < 0.001). Similarly, the infiltration level of M2-polarized (CD206 +) TAMs was significantly increased in BMC tissues of T3-4 (P = 0.004) and N + (P = 0.009). In addition, the infiltration level of M2-polarized (CD206 +) TAMs was confirmed to be associated with advanced staging (P = 0.001). In addition, we used the multivariate logistic regression to identify the independent risk factors of M2-polarized TAMs’ infiltration level. However, the results of multivariate logistic regression showed that none of these variables were independent risk factors of the infiltration level of M2-polarized TAMs (Table S1, P < 0.05). Moreover, the infiltration level of M2-polarized TAMs was not related to the pathological grading of BMC patients in this study (P > 0.05).

**Table 2:** Correlation analysis between infiltration of M2-polarized TAMs and clinical characteristics

Pathological features	Expression of M2-polarized (CD68+) TAMs		χ2	P-value	Expression of M2-polarized (CD163 +) TAMs		χ2	P-value	Expression of M2-polarized (CD206 +) TAMs		χ^2^	P-value
	Low	High			Low	High			Low	High		
Gender			4.381	0.036			4.016	0.045			1.095	0.295
Female	0	4			0	4			1	3		
Male	23	19			22	20			22	20		
Age			0.348	0.555			0.809	0.369			1.394	0.238
< 49 years old	10	12			9	13			9	13		
≥ 49 years old	13	11			13	11			14	10		
T stage			0.168	0.681			7.568	0.006			8.256	0.004
T1–T2	20	19			22	17			23	16		
T3–T4	3	4			0	7			0	7		
N stage			0.138	0.71			6.045	0.014			6.769	0.009
N0	19	18			22	12			22	15		
N1–N3	4	5			0	12			1	8		
Clinical stage			0.451	0.502			14.882	< 0.001			11.275	0.001
I–II	18	16			18	16			22	12		
III–IV	5	7			5	7			1	11		
Pathological grading			0.965	0.326			0.637	0.425			0.965	0.326
Well differentiated	5	8			5	8			8	5		
Poorly differentiated	18	15			17	16			15	18		

### Expression of PD-L1, CD8+ T cells, and EGFR in BMC

For investigating the expression level of PD-L1 in BMC cells and tumour infiltrating lymphocytes (TILs), we used TPS, IPS, and CPS for evaluation (Table 3). In BMC cells, PD-L1 positivity was observed in 25 BMC patients, with a TPS cut-off value of ≥ 1%. In contrast, TILs of 31 (67.4%) patients stained positive at IPS cut-off values of ≥ 1%. Furthermore, 33 (71.7%) patients had CPS ≥ \1. According to the results of immunohistochemistry, the median density of CD8+ T cells was 25%, and 52.2% (24/46) showed high infiltration in the tumour area. In total, 0.0% (0/46) of samples had negative EGFR expression, and 100% (46/46) had high EGFR expression; among the positive samples, 45.7% (21/46) had low EGFR expression, and 54.3% (25/46) had high EGFR expression. EGFR was expressed in the BMC tumour area in 100% (46/46) of samples; 45.7% (21/46) of samples had low expression, and 54.3% (25/46) of samples had high expression.

**Table 3:** Expression of PD-L1, CD8+ T cells, and EGFR in BMC

	Tumor tissues	Paracancerous tissues
PD-L1-TPS		
−	47.8% (22/46)	
+	52.2% (25/46)	
PD-L1-IPS		
−	32.6% (15/46)	78.3% (36/46)
+	67.4% (31/46)	21.7% (10/46)
PD-L1-CPS		
−	28.3% (13/46)	
+	71.7% (33/46)	
CD8+ T cells infiltration		
−	0.0% (0/46)	8.7% (4/46)
Few	47.8% (22/46)	65.2 (30/46)
Massive	52.2% (24/46)	26.1 (12/46)
Expression of EGFR		
None	0.0% (0/46)	45.7% (21/46)
Low	45.7% (21/46)	52.2% (24/46)
High	54.3% (25/46)	2.2% (1/46)

### The correlation between TAM infiltration and PD-L1 expression

**Fig. 5:**
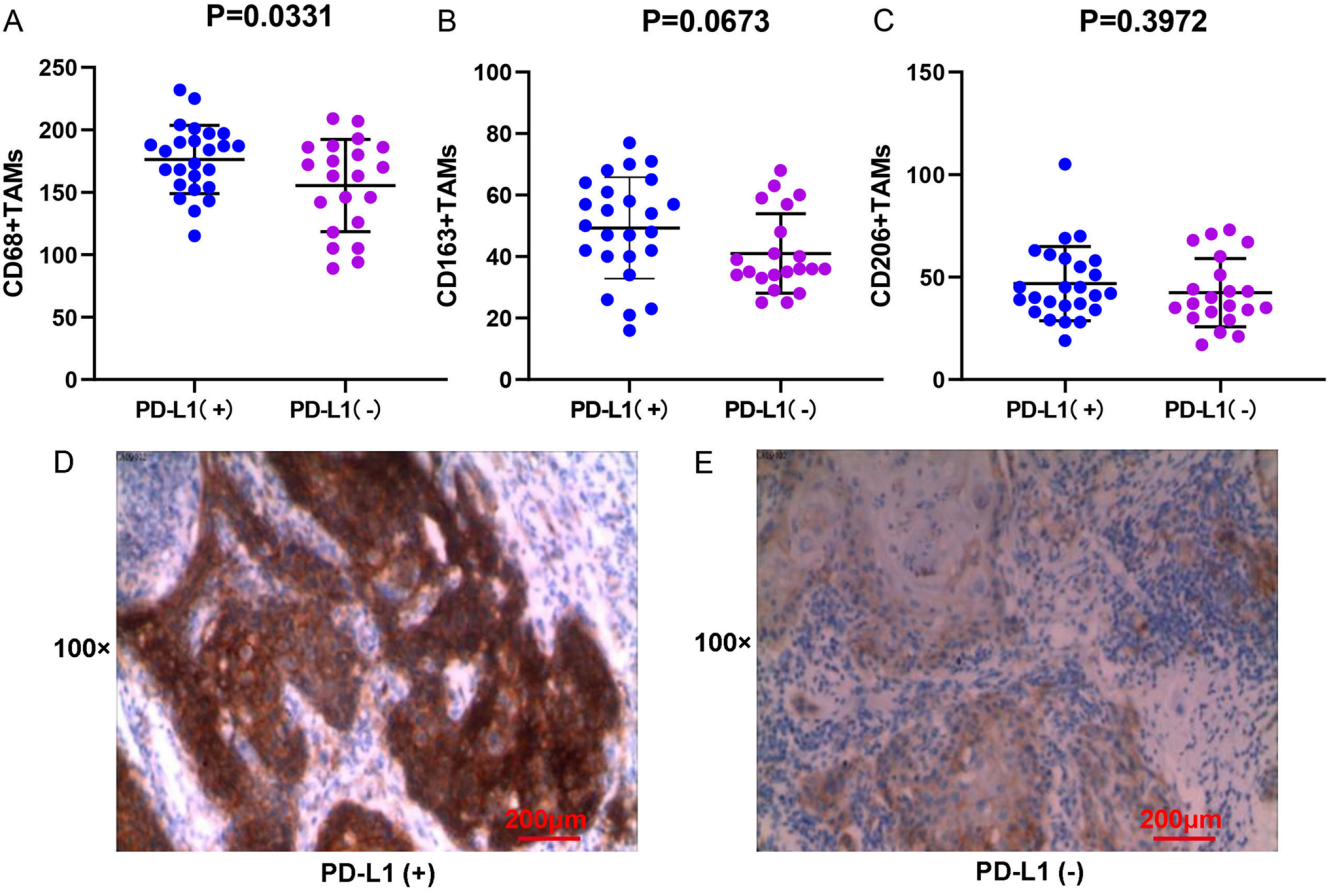
Correlation between PD-L1 TPS score and M2-polarized TAMs infiltration in BMC. **A** M2-polarized (CD68+) TAMs infiltration and the PD-L1 TPS. **B** M2-polarized (CD163+) TAMs infiltration and the PD-L1 TPS. **C** M2-polarized (CD206+) TAMs infiltration and the PD-L1 TPS. **D** IHC staining result of BMC tumor cells with high expression of PD-L1. **E** IHC staining result of BMC tumor cells with low expression of PD-L1. Scale bar = 200 μm

**Fig. 6:**
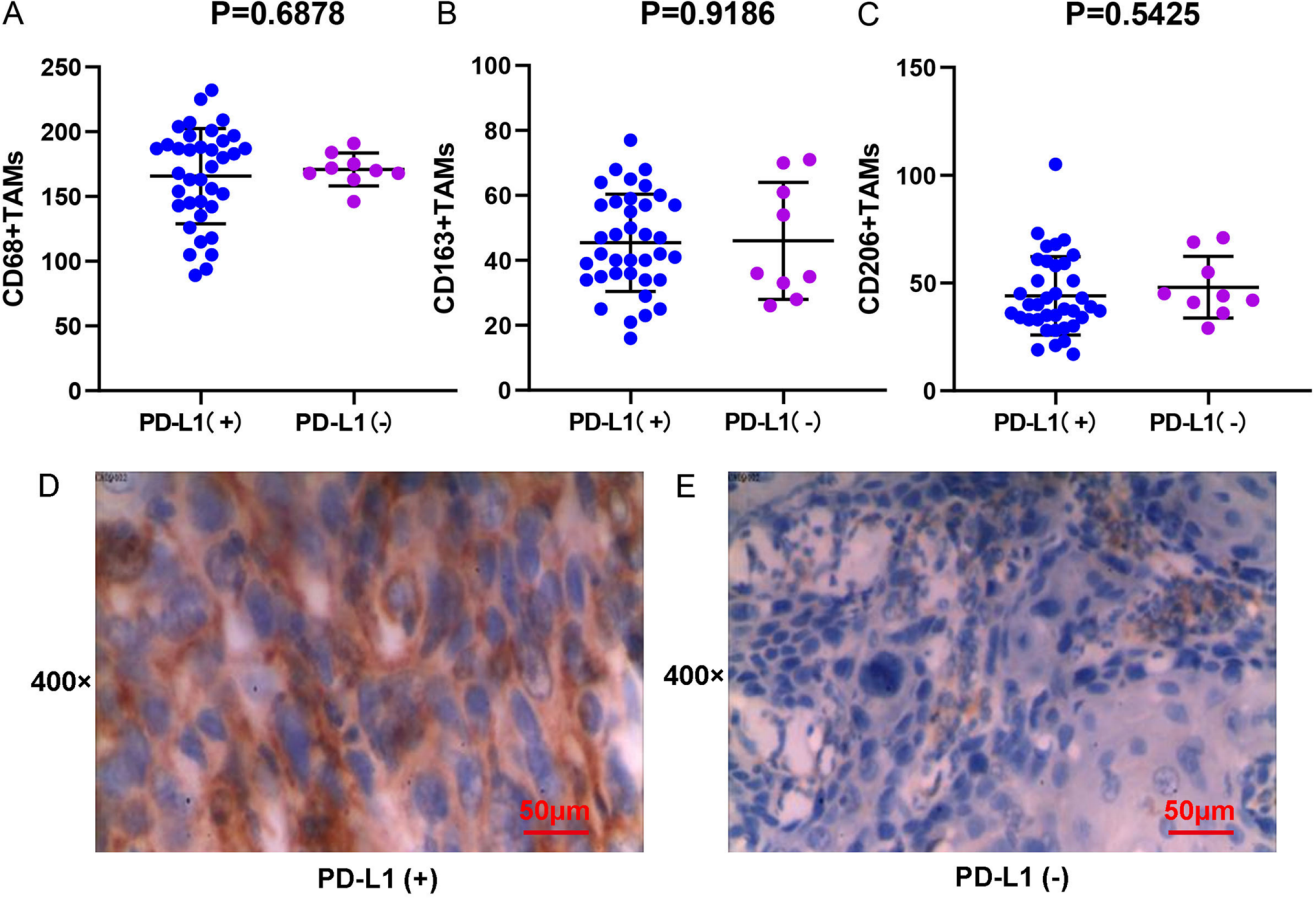
Correlation between the expression of PD-L1 in tumor infiltrating immune cells and the M2-polarized TAMs infiltration in BMC. **A** M2-polarized (CD68+) TAMs infiltration and the PD-L1 IPS. **B** M2-polarized (CD163+) TAMs infiltration and the PD-L1 IPS. **C** M2-polarized (CD206+) TAMs infiltration and the PD-L1 IPS. **D** IHC staining result of BMC tumor infiltrating immune cells with high expression of PD-L1. **E** IHC staining result of BMC tumor infiltrating immune cells with low expression of PD-L1. Scale bar = 50 μm

**Fig. 7:**
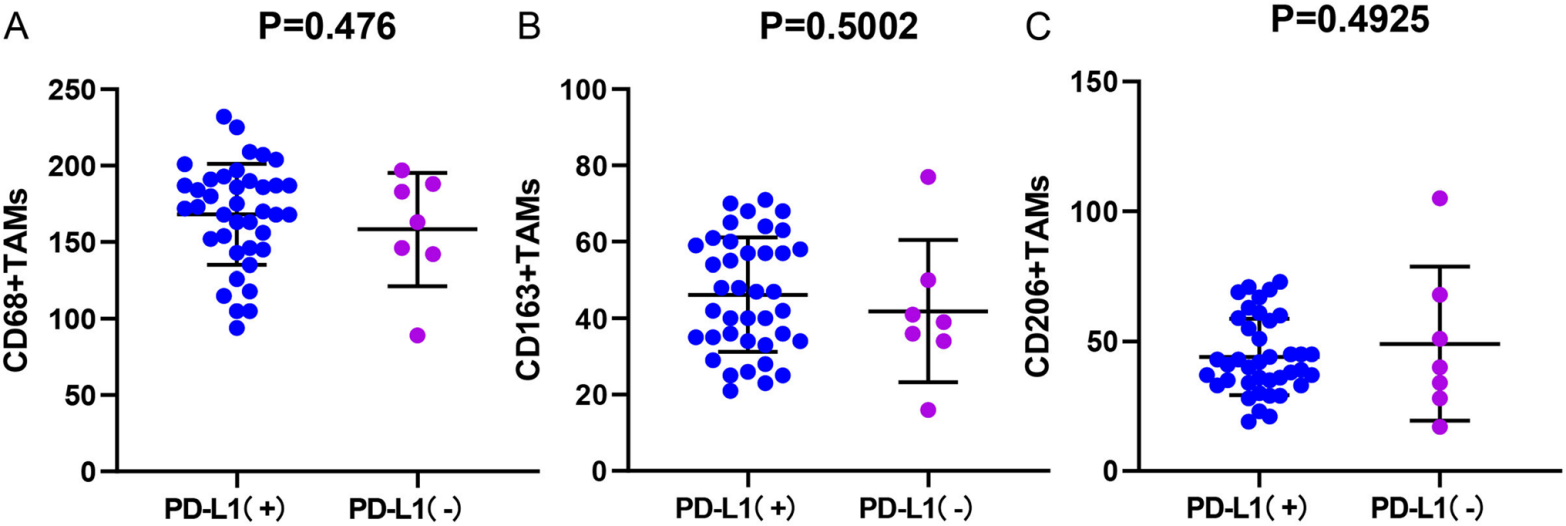
Correlation between PD-L1 CPS score and M2-polarized TAMs infiltration in BMC. **A** M2-polarized (CD68+) TAMs infiltration and the PD-L1 CPS. **B** M2-polarized (CD163+) TAMs infiltration and the PD-L1 CPS. **C** M2-polarized (CD206+) TAMs infiltration and the PD-L1 CPS

As shown in Fig. 5A, the infiltration level of M2-polarized (CD68+) TAMs (P = 0.0331) in the PD-L1 TPS positive group (Fig. 5D) was significantly increased compared to that in the PD-L1 TPS negative group (Fig. 5E). However, in the high PD-L1 TPS, IPS, and CPS groups, there was no significant increase in infiltration of M2-polarized (CD163 + and CD206 +) TAMs in BMC tissues (Figs. 5B, C, 6, 7, P > 0.05).

### Correlation analysis between TAM infiltration and CD8+ T-cell infiltration

Chi-square test showed that there was no significant correlation between M2-polarized (CD68+, CD163+ and CD206+) TAMs infiltration and CD8+ T lymphocyte infiltration (Table 4, P > 0.05).

**Table 4:** Correlation between CD8+ T cells and infiltration of M2-polarized TAMs

	CD8+ T cells infiltration		χ^2^	P-value
	Few	Massive		
M2-polarized (CD68+) TAMs infiltration			1.394	0.238
Few	13	10		
Massive	9	14		
M2-polarized (CD163 +) TAMs infiltration			2.144	0.143
Few	13	9		
Massive	9	15		
M2-polarized (CD206 +) TAMs infiltration			3.136	0.077
Few	14	9		
Massive	8	15		

### Relationship between EGFR expression and TAMs infiltration

The infiltration level of M2-polarized (CD206+) TAMs in the high EGFR expression group was much higher than that in the low EGFR expression group (Table 5, P < 0.05). There was no significant correlation between the infiltration level of M2-polarized (CD163+ and CD68+) TAMs and the expression of EGFR (P > 0.05).

**Table 5:** Correlation between EGFR and infiltration of M2-polarized TAMs

	Expression of EGFR		χ^2^	P-value
	Low	High		
M2-polarized (CD68+) TAMs infiltration			0.383	0.54
Few	9	14		
Massive	7	16		
M2-polarized (CD163+) TAMs infiltration			0.088	0.77
Few	11	10		
Massive	12	13		
M2-polarized (CD206+) TAMs infiltration			4.293	0.04
Few	14	7		
Massive	9	16		

## Discussion

To sum up, our above study investigated the correlation between the infiltration level of M2-polarized TAMs in BMC and clinical pathological factors (including PD-L1 expression, EGFR expression and CD8+ T cell levels). The results showed that M2-polarized TAMs infiltration increased in BMC tissues, and the degree of M2-polarized TAM infiltration in female samples was significantly higher than that in male samples. The degree of infiltration of M2-polarized TAMs is positively associated to the T stage, N stage, and clinical stage of BMC patients. The infiltration of M2-polarized TAMs was positively correlated with the expression of PD-L1 and EGFR. In addition, our study found M2-polarized TAMs can serve as a potential diagnostic biomarker in BMC.

Previous research showed that increased infiltration of M2-polarized TAMs was associated with the development of oral cancer in oral leukoplakias and related to malignant transformation [23]. In addition, some studies have reported that the buccal mucosa is the most common site of oral cancer in South Asians [24, 25], which may be related to smoking and betel nut chewing habits [26, 27]. Zhou et al. found that low-dose arecoline promotes the proliferation of fibroblasts and OSCC cells by accelerating cell cycle progression [28]. However, BMC has often been analysed together with other oral cancers, making it impossible to obtain specific conclusions about BMC [29, 30], so it is necessary to conduct further independent research on BMC and M2-polarized TAMs.

As a significant component of the tumour microenvironment (TME), the metabolic changes of TAMs are associated with the promotion of malignant cancer progression [31], and TAMs can support the proliferation, invasion, metastasis and angiogenesis of cancer [32, 33], which is associated to the poor outcomes of malignant cancer patients [34, 35]. For example, the increased infiltration of M2-polarized TAMs is correlated with the progression of prostatic adenocarcinoma [36], liver cancer [37], colorectal cancer [38] and breast cancer [39]. However, the difference in M2-polarized TAMs infiltration between BMC samples and non-BMC samples has not been comprehensively reported before.

In present study, we revealed the upregulation of M2-polarized TAMs markers (CD68, CD163, CD206) in BMC tissues with IHC, which was consistent with the results of previous studies. In terms of the clinical characteristics of M2-polarized TAMs, Fan Guo et al. indicated that the level of M2-polarized (CD68+) TAMs infiltration was related to the N stage of cervical carcinoma, and the M2-polarized (CD163+) TAMs density was related to lymph node metastasis and FIGO stage [40].

In terms of gender, our study found that gender is a risk factor for the infiltration level of M2-polarized (CD68+ and CD163+) TAMs in BMC, and the infiltration level of females is higher than that of males. However, this finding is contrary to previous studies in non-small cell lung cancer and liver cancer [41, 42], which may be related to the small size of female samples collected. Therefore, a larger cohort is needed to verify the performance of M2-polarized TAMs infiltration level between gender in BMC patients.

In addition, high infiltration of M2-polarized (CD206+ and CD163+) TAMs was correlated with larger tumour size, lymph node metastasis, and more advanced clinical stage of BMC. Ye et al. showed that M2-polarized (CD163+) TAMs inhibit phagocytosis and affect the secretion of IL-6 and tumor necrosis factor-α (TNF-α), as well as IL-10 and TGF-β, by downregulating the expression of signal regulatory protein α (SIRPα), thereby promoting the process from normal to oral potentially malignant diseases (OPMDs) and then to OSCC [43]. IL-10 had also been found to be involved in the M2 macrophage polarization process in oral cancer [44]. In summary, we speculate that IL-10 may act as a key molecule to promote the occurrence of OSCC. In addition, previous studies have shown that IL-10 secreted by M2 TAMs plays an important role in the growth of various tumors, such as prostate cancer and intrahepatic cholangiocarcinoma [45, 46]. In summary, this indicated that it’s worth further exploring the role of IL-10 in M2-polarized TAMs promoting the progression of BMC in the future. Apart from above potential mechanisms, Wang et al. also found that M2-polarized TAMs may promote metastasis of OSCC by generating stress granule overexpressing CCL3 [47]. The above findings indicated that the infiltration of M2-polarized TAMs may be associated with the progression of BMC.

Furthermore, in order to explore the probability of targeting M2-polarized TAMs in combination with immunotherapy, we also investigated the correlation between M2-polarized TAMs and PD-L1, EGFR, and CD8+ T cells. As an oncogenic gene, EGFR is considered one of the targets for precise treatment of multiple malignant cancers with EGFR mutations [48, 49], and has been found to be associated with a poor prognosis in various malignant cancers [50–52]. Based on many reliable clinical trials, multiple PD-L1 antibodies have been widely used for treating malignant tumours [53]. Thus, it is worthwhile to explore the correlation between M2-polarized TAMs and EGFR/PD-L1 in BMC patients. Our study found that the expression/infiltration of PD-L1, EGFR, and CD8+ T cells was increased in BMC tumour tissues vs. normal tissues. We also found a significant increase in the expression of an M2-polarized TAM marker (CD68+) in the high PD-L1 TPS group and a significant increase in the expression of another M2-polarized TAMs marker (CD206+) in the high EGFR expression group.

It was reported that M2-polarized (CD206+) TAMs might play a momentous role in the progression of OSCC and ovarian cancer by secreting EGF [54, 55], and PD-L1 expression was increased in M2-polarized (CD68+) TAMs [56, 57], indicating that high infiltration of M2-polarized (CD206+) TAMs in BMC tissues may become a predictive marker for the response to EGFR-targeted treatment, while the high infiltration of M2-polarized (CD68+) TAMs may be an indicator of immunotherapy response. Zhao et al. thought that M2-polarized TAMs may induce EMT in cancer cells by activating the EGFR/ERK1/2 signaling pathway for head and neck squamous cell carcinoma progression [58]. Moreover, PD-L1 has been indicated to be highly expressed in TAMs and closely associated with tumor immunotherapy resistance [59, 60]. Based on the above research, abnormal infiltration level of M2-polarized TAMs may influence the tumorigenesis of BMC by regulating the expression levels of EGFR and PD-L1. One study identified that the deficiency of N6-methyladenosine methyltransferase Mettl14 in TAMs can inhibit CD8+ T-cell infiltration and promote tumour growth [61]. Unfortunately, our study did not find a significant relationship between the expression of TAMs in BMC tissues and the degree of CD8+ T-cell infiltration. This result may be related to a small sample size and single experimental method.

Overall, our current work applied IHC to detect the infiltration level of M2-polarized TAMs in BMC and further explored the clinicopathological significance of M2-polarized TAMs. In addition, this study found that the expression of PD-L1 and EGFR was related to the infiltration of M2-polarized TAMs. Experimental studies are needed to elucidate the mechanism by which M2-polarized TAMs promote the progression of BMC. Single cell RNA sequencing analysis technology can be used to identify the differential genes of M2-polarized TAMs in normal tissues and BMC tissues, and to verify their role in promoting BMC progression through in vitro and in vivo functional experiments. M2-polarized TAMs are widely recognized as associated with poor prognosis in malignant tumors, and the transformation of TAMs from M1 into M2 is considered a new target for tumor treatment. Therefore, it is important to explore the key genes that can promote the transformation of M2-polarized TAMs into M1-polarized TAMs in the future. Moreover, correlation analysis can only provide preliminary evidence of the correlation rather than determine the potential mechanism between clinical pathological parameters and M2-polarized TAMs. For revealing the mechanism between clinical pathological parameters and M2-polarized TAMs, experimental research and large-scale sample collection are still needed.

## Conclusion

Collectively, this study revealed the high expression and clinicopathological significance of M2-polarized TAMs in BMC by IHC for the first time. Based on the clinicopathological features of BMC patients, we found that a high level of M2-polarized TAMs infiltration might accelerate the progression of BMC. In addition, we found that the infiltration level of M2-polarized TAMs was positively correlated with the expression of PD-L1 and EGFR, which suggests that M2-polarized TAMs can be used as a critical target for the treatment of BMC. However, more samples are required to determine the prognosis significance of M2-polarized TAMs, and more experiments are needed to reveal the underlying mechanisms of M2-polarized TAMs.

### Supplementary Information

Supplementary Material 1.

## Data Availability

The datasets used and analysed during the current study available from the corresponding author on reasonable request.
