# Supplementary material for: Increased infiltration of M2-polarized tumour-associated macrophages is highly associated with advanced disease stage and high expression of PD-L1 in buccal mucosa carcinoma
Source: Discov Oncol. 2024 Jul 29;15:314. doi: 10.1007/s12672-024-01190-y (PMC11286931; doi:10.1007/s12672-024-01190-y)
Supplement: Supplementary file 1 — Supplementary Material 1. [file 12672_2024_1190_MOESM1_ESM.docx]

**Table S1.** Multivariate logistic regression on the infiltration level of M2-polarized TAMs in BMC based on clinical features.

| **Pathological features** | | **Expression of M2-polarized (CD68+) TAMs** | | | **Expression of M2-polarized (CD163+) TAMs** | | | **Expression of M2-polarized (CD206+) TAMs** | | |
| --- | --- | --- | --- | --- | --- | --- | --- | --- | --- | --- |
|  |  | **OR** | **95% CI** | **p value** | **OR** | **95% CI** | **p value** | **OR** | **95% CI** | **p value** |
| **Gender** | |  |  |  |  |  |  |  |  |  |
|  | female | Reference | | | Reference | | | ———— | | |
|  | male | 0.303 | 0.029-3.155 | 0.318 | 1.243 | 0.129-11.957 | 0.85 |  |  |  |
| **T stage** | |  |  |  |  |  |  |  |  |  |
|  | T1-T2 | ———— | | | Reference | | | Reference | | |
|  | T3-T4 |  |  |  | 0.067 | 0.003-1.571 | 0.0093 | 0.223 | 0.016-3.272 | 0.276 |
| **N stage** | |  |  |  |  |  |  |  |  |  |
|  | N0 | ———— | | | Reference | | | Reference | | |
|  | N1-N3 |  |  |  | 0.671 | 0.044-10.124 | 0.773 | 0.499 | 0.034-7.305 | 0.612 |
| **Clinical stage** | |  |  |  |  |  |  |  |  |  |
|  | I-II | ———— | | | Reference | | | Reference | | |
|  | III-IV |  |  |  | 8.71 | 0.293-259.207 | 0.211 | 1.935 | 0.104-35.885 | 0.658 |
